# Etiology of Dental Caries

**Published:** 1883-03

**Authors:** 


Etiology of Dental Caries.—Last month we published a paper by Dr. Stockwell, upon this subject. This month we give another by Dr. Clark, both in support of the germ theory of decay. Next month we shall print one by Prof. Frank Abbott, in support of the vital theory, or the inflammatory hypothesis. Our May number will contain yet another paper, by Dr. W. D. Miller, of Berlin, presenting his theory of dental caries. The June number will contain an article giving the views of those who support the chemical theory, prepared for us by one who is an acknowledged authority.

It will be seen that all these papers are written by representative men, and we candidly believe that it will be the most valuable series of essays ever presented to the profession upon this subject. We hope they will be as widely read as their great importance demands.

Intending subscribers who desire to possess all of the series should have their subscription begin with the year.

